# Glycogen Metabolism Predicts the Efficacy of Immunotherapy for Urothelial Carcinoma

**DOI:** 10.3389/fphar.2021.723066

**Published:** 2021-08-25

**Authors:** Yueming Zhang, Xuechun Li, Rui Zhou, Anqi Lin, Manming Cao, Qingwen Lyu, Peng Luo, Jian Zhang

**Affiliations:** ^1^ Department of Oncology, Zhujiang Hospital, Southern Medical University, Guangzhou, China; ^2^ Department of Information, Zhujiang Hospital, Southern Medical University, Guangzhou, China

**Keywords:** urothelial cancer, glycogen metabolism, immunotherapy, prognosis, gene mutation, immune microenvironment, pathway activation, drug sensitivity

## Abstract

Urothelial cancer (UC) is one of the common refractory tumors and chemotherapy is the primary treatment for it. The advent of immune checkpoint inhibitors (ICI) has facilitated the development of treatment strategies for UC patients. To screen out UC patients sensitive to ICI, researchers have proposed that PD-L1, tumor mutation burden and TCGA molecular subtypes can be used as predictors of ICI efficacy. However, the performance of these predictors needs further validation. We need to identify novel biomarkers to screen out UC patients sensitive to ICI. In our study, we collected the data of two clinical cohorts: the ICI cohort and the TCGA cohort. The result of the multivariate Cox regression analysis showed that glycogen metabolism score (GMS) (HR = 1.26, *p* = 0.017) was the negative predictor of prognosis for UC patients receiving ICI treatment. Low-GMS patients had a higher proportion of patients achieving complete response or partial response to ICI. After the comparison of gene mutation status between high-GMS and low-GMS patients, we identified six genes with significant differences in mutation frequencies, which may provide new directions for potential drug targets. Moreover, we analyzed the immune infiltration status and immune-related genes expression between high-GMS and low-GMS patients. A reduced proportion of tumor-associated fibroblasts and elevated proportion of CD8^+^ T cells can be observed in low-GMS patients while several immunosuppressive molecules were elevated in the high-GMS patients. Using the sequencing data of the GSE164042 dataset, we also found that myeloid-derived suppressor cell and neutrophil related signature scores were lower in α-glucosidase knockout bladder carcinoma cells when compared to the control group. In addition, angiogenesis, classic carcinogenic pathways, immunosuppressive cells related pathways and immunosuppressive cytokine secretion were mainly enriched in high-GMS patients and cell samples from the control group. Finally, we suspected that the combination treatment of ICI and histone deacetylase inhibitors may achieve better clinical responses in UC patients based on the analysis of drug sensitivity data. In conclusion, our study revealed the predictive value of GMS for ICI efficacy of UC patients, providing a novel perspective for the exploration of new drug targets and potential treatment strategies.

## Introduction

Urothelial cancer (UC) is one of the most common refractory tumors of the genitourinary system. It is comprised of a group of tumors occurring in several regions like the renal pelvis, ureter, bladder, and urethra. Notably, bladder urothelial cancer accounts for over 90% of UC cases annually (Milojevic et al., 2015). According to GLOBOCAN 2020 statistics released by the International Agency for Research on Cancer, there were approximately 573,000 new cases of bladder cancer globally in 2020 (Sung et al., 2021). These cases accounted for 3% of new cancer cases worldwide and 2,13,000 deaths resulting from bladder cancer (Sung et al., 2021). Among them, patients with metastatic urothelial cancer (mUC) have the worst prognosis, with a median overall survival (OS) after receiving chemotherapy of about 15 months (Mollica et al., 2020). The 5 year survival rate of mUC patients is only 6.4%, and while that of patients with carcinoma *in situ* is 96% (SEER*Explore, 2021).

Over the last 30 years, the treatment of urothelial cancer has remained unchanged, with platinum-based combined chemotherapy being largely considered the cornerstone of the treatment of UC patients (Mollica et al., 2020). However, since its advent in 2016, immunotherapy has revolutionized the treatment of UC patients. Five immune checkpoint inhibitors (ICI) have been approved by the US Food and Drug Administration (FDA) for application in UC patients (Mollica et al., 2020). KEYNOTE-045 (Bellmunt et al., 2017), IMvigor211 (Powles et al., 2018), and IMvigor130 (Galsky et al., 2020) trials have shown that compared with chemotherapy, ICI monotherapy can achieve durable remission for UC patients with an improved OS of about 3 months. In addition, IMvigor130 trial also explored the use of atezolizumab in combination with chemotherapy, with results suggesting a significant prolongation of median OS in UC patients receiving combination therapy when compared to patients who received chemotherapy (Galsky et al., 2020).

Currently, ICIs have been used as the first-line treatment for PD-L1 positive and cisplatin-ineligible UC patients (Mollica et al., 2020). However, durable responses only occurred in 20–30% of UC patients (Nadal and Bellmunt, 2019). The realization of an accurate prediction of patients sensitive to ICI is a common research challenge. In addition to the expression of immune checkpoint molecules, the exploration of biomarkers for ICI treatment has focused on tumor mutation burden (TMB) (Rosenberg et al., 2016; Powles et al., 2018), microsatellite instability (MSI) (Lin et al., 2020a), gender (Lin et al., 2020b), age (Lin et al., 2020b), gene mutations (Lin et al., 2019; Lin et al., 2020b; Huang et al., 2020; Niu et al., 2020; Yi et al., 2020; Zhang et al., 2021) and alterations of the immune microenvironment (Lin et al., 2020a). Some proposed biomarkers for the prediction of the ICI efficacy in patients with mUC include PD-L1 (Rosenberg et al., 2016; Balar et al., 2017; Powles et al., 2017; Sharma et al., 2017), TMB (Rosenberg et al., 2016; Powles et al., 2018), TCGA molecular subtypes (Rosenberg et al., 2016; Sharma et al., 2017), and the expression of immune-related genes such as IFN-γ (Sharma et al., 2017). However, it is still difficult to achieve accurate predictions for these indicators. In the IMvigor210 and CheckMate-275 trials, the study on PD-L1’s predictive effect on the efficacy of the ICI did not obtain statistically significant results (Rosenberg et al., 2016; Sharma et al., 2017). Similarly, the outcomes of the JAVELIN Solid Tumor clinical trial did not conclude that TMB was related to the efficacy of ICI in UC patients (Patel et al., 2018). The IMvigor210 trial concluded that patients with TCGA luminal cluster II subtype had the highest objective response rate (Rosenberg et al., 2016). In contrast, the results of the CheckMate-275 trial suggested that patients with basal I subtype exhibited the most favorable prognosis (Sharma et al., 2017). Therefore, identifying new and specific biomarkers to screen out patients with better ICI responses has become an urgent problem to be solved in the treatment of urothelial carcinoma.

In the tumor microenvironment (TME), glycogen metabolism profoundly affects tumor cell proliferation and migration and has a significant regulatory effect on immune cells. Dendritic cell activation, as well as macrophage function and CD8^+^ T cell survival also depend on glycogen metabolism (Khan et al., 2020). There is competition for glucose uptake between tumor and immune cells. In addition, studies have shown that reducing the glycogen branching enzyme could promote CD8^+^ T cell infiltration and increase PD-L1 expression (Li et al., 2019). Inhibition of glycogen synthase kinase (GSK) three can promote NK cell maturation and enhance their anti-tumor activity (Cichocki et al., 2017). GSK3 is also a key upstream kinase that regulates PD-1 transcription in T cells (Sahin et al., 2019). The role of glycogen metabolism in tumor and immune cells suggests that it plays a critical role in the immunotherapy of UC patients.

In this study, we explored the effect of glycogen metabolism on immunotherapy efficacy in mUC patients using the ICI cohort’s data. We also investigated the impact of glycogen metabolism on the tumor immune microenvironment from the aspects of immune-related cell content, immune gene activation, tumor immune depletion indicators, and abnormal pathway activation. To further clarify the impact of glycogen metabolism on the immune infiltration landscape, we also quantified alterations in the TME-related signatures using GEO dataset containing sequencing data for bladder carcinoma cells accepting different intervention on glucose metabolism. Finally, we use the drug sensitivity data in public databases to predict the activity of various drugs in UC patients, which facilitates further exploration for potential treatment strategies.

## Materials and Methods

### Clinical Cohorts

In this study, we collected data on two clinical cohorts and the GSE164042 dataset from the GEO database to explore the impact of glycogen metabolism on the immune microenvironment of UC patients. One of the two clinical cohorts is the ICI cohort published by Mariathasan et al. (Mariathasan et al., 2018), which is comprised of 348 mUC patients treated with atezolizumab. The other clinical cohort is comprised of 408 bladder cancer patients collected from the TCGA database.

RNA sequencing (RNA-seq) data, somatic mutation data, TMB data, tumor neoantigen load (TNB) data, and corresponding clinical data are available for both clinical cohorts. The processed ICI cohort data can be obtained from the R package “IMvigor210CoreBiologies” (Mariathasan et al., 2018), available at http://research-pub.gene.com/IMvigor210CoreBiologies. The TCGA cohort’s data were downloaded from the Genomic Data Commons Data Portal (https://portal.gdc.cancer.gov/). TMB refers to the total number of mutations per megabase in the exon coding region of tumor genome. The prediction methods for TNB have been described in the corresponding literatures (Mariathasan et al., 2018; Thorsson et al., 2018).

**FIGURE 1:**
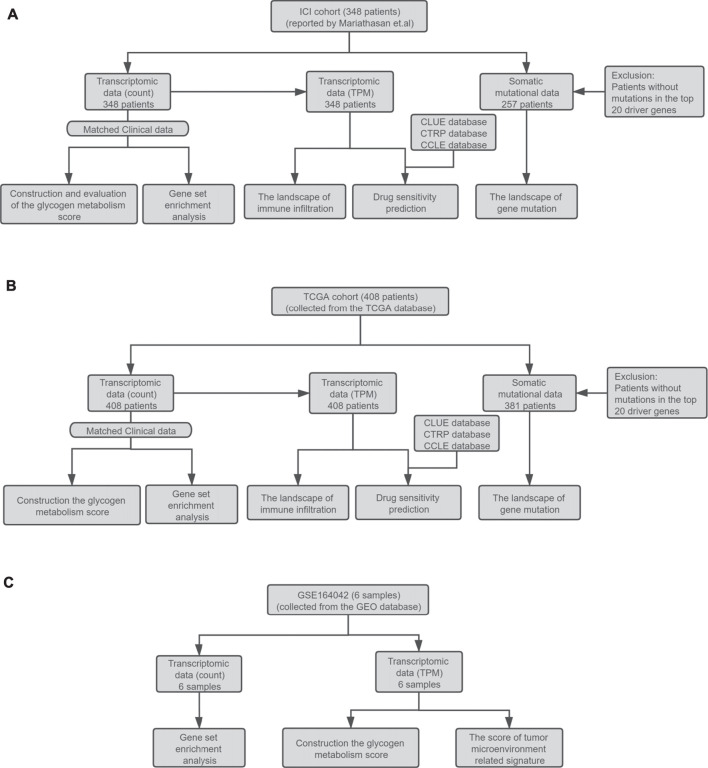
**(A)** Establishment of the ICI cohort. The ICI cohort published by Mariathasan et al. included 348 UC patients who received the treatment of atezolizumab. **(B)** The establishment of the TCGA cohort. The TCGA cohort contained 408 bladder cancer patients collected from the TCGA database. The RNA-seq count data, somatic mutation data, and corresponding clinical data of the two cohorts were then used to evaluate the prognostic value of GMS, explore the landscape of gene mutation and immune infiltration, GSEA analysis, and drug sensitivity prediction. **(C)** The collection of the high throughput sequencing data of GSE164042. This dataset contained six samples from the UMUC3 bladder carcinoma cell line. The RNA-seq data and corresponding grouping information of the samples were used to construct the GMS scores and tumor microenvironment related signature scores and perform GSEA analysis.

In addition, the RNA expression level of the same patient is represented by the mean of the RNA expression levels in multiple samples. The RNA-seq read counts were then normalized in the two cohorts, and transcripts per kilobase of exon model per million mapped reads (TPM) were used to quantify the RNA expression level. The detailed analysis workflow is shown in Figure 1.

The GSE164042 dataset contains high-throughput sequencing data of six UMUC3 bladder carcinoma cell samples, three from the control group (GEO-control) and three from the α-glucosidase II knockout group (GEO-KO). The RNA-seq count data and normalized TPM data can be downloaded from the GEO database, and the detailed analysis workflow is shown in Figure 1.

### The Predictive value of the Glycogen Metabolism Pathway

In this study, we used a single sample gene set enrichment analysis (ssGSEA) algorithm (Barbie et al., 2009) to evaluate each patient’s glycogen metabolism pathway enrichment scores (WP_GLYCOGEN_SYNTHESIS_AND_DEGRADATION), and UC patients were divided into two groups, high glycogen metabolism and low glycogen metabolism, according to their median scores. In addition, we also evaluated the glycogen metabolic pathway scores of 6 cell samples using RNA-seq data from the GSE164042 dataset.

To evaluate the predictive value of the glycogen metabolism enrichment score, we incorporated the ICI cohort’s clinical information in the univariate COX regression model. The indicators with potential predictive significance for the UC patients’ ICI efficacy were also included in the analysis. The statistically significant indicators (*p* < 0.05) were included in the multivariate COX regression model. After excluding two patients with unqualified survival data in the TCGA cohort, we used Kaplan-Meier analysis and log-rank test to evaluate the survival differences of patients in the two glycogen metabolism groups.

### Evaluation of Immune Cell Infiltration and the Expression of Immune-Related Genes

EPIC is a deconvolution algorithm that can obtain the proportions of the cell types by analyzing the expression levels of the marker genes of different immune cells (Racle and Gfeller, 2020). In addition, this algorithm’s principle, performance, and workflow have been described in the literature. In this study, we used standardized TPM expression data as our input, and this algorithm was used to quantify each patient’s immune infiltration. *p* < 0.05 was considered statistically significant. The R package which executes this algorithm can be found at https://github.com/GfellerLab/EPICobtain.

The list of immune-related genes of the TCGA cohort was published by Thorsson et al. (2018). R package “edgeR” was utilized to calculate the logFC and *p*-value of gene differential expression (Robinson et al., 2010). According to the list of immune-related genes and their functional classification, we extracted their logFC and *p*-value for visualization.

Since the EPIC algorithm cannot be used to assess the degree of immune infiltration in cell lines, we used the PCA algorithm to assess the scores of 119 TME-related signatures provided by “IOBR” package for UC patients and cell samples (Zeng et al., 2021). R package “IOBR” is available at https://github.com/IOBR/IOBR.

### Functional and Pathway Enrichment Analysis

The R package “edgeR” was used to standardize the RNA-seq count data and perform gene differential expression analyses (Robinson et al., 2010). The genes were sorted in decreasing order of logFC derived from differential analyses. The R package, “clusterProfiler” (Yu et al., 2012), was employed for gene set enrichment analysis (GSEA), using the sorted gene lists and gene sets collected from the Molecular Signatures Database (MSigDB) of Broad Institute (https://www.gsea-msigdb.org/gsea/msigdb/index.jsp) (Subramanian et al., 2005). Gene sets used were as follows: C2 curated gene sets, C5 ontology gene sets, C6 oncogenic signature gene sets, and C7 immunologic signature gene sets.

### Prediction of Drug Sensitivity

The CLUE database primarily contains gene expression profiles and corresponding drug sensitivity information after different perturbagens (small molecule treatment, gene overexpression, or gene knockout) on different cell lines, accessible through https://clue.io/. We uploaded the differentially expressed genes between patients with high and low glycogen metabolism. Then, we analyzed and obtained the degree of similarity between the expression profiles of our clinical cohorts and that of cell lines receiving different drug treatments. The similarity was quantified with a score of −100 to 100, where a positive value represents a similar trend, and a negative value represents an opposite trend. Additionally, the higher absolute value of the score represents a more obvious trend. We screened out the drugs with absolute scores above 60 for follow-up evaluation. Their mechanism of action can be obtained in the Touchstone module of the CLUE database.

The Cancer Therapeutics Response Portal (CTRP, v2 version) database contains the drug sensitivity data of 481 compounds in 860 cancer cell lines, which can be accessed at https://portals.broadinstitute.org/ctrp.v2.1/. The CTRP database uses the area under the dose-response curve (area under the curve, AUC) to measure drug sensitivity. Observation revealed that lower AUC values correlated with a higher sensitivity to the drug.

The Cancer Cell Line Encyclopedia (CCLE) database contains data on genetic mutations, RNA splicing, DNA methylation, histone modification, miRNA expression, and protein expression data of 1,457 cell lines, which can be accessed at https://portals.broadinstitute.org/ccle/(Ghandi et al., 2019).

Using drug sensitivity data from the CTRP database and the CCLE database’s cancer cell line expression profiles, we employed the R package “pRRophetic” to predict the drug sensitivity of this study’s clinical cohorts (Geeleher et al., 2014). We excluded the data of hematological tumors and compounds with more than 20% missing data before prediction and used k-nearest neighbor (k-NN) imputation to fill in missing AUC values. Finally, we analyzed the differences in the AUC values between patients with high and low glycogen metabolism and calculate the correlation between AUC values and the glycogen metabolism scores. The drugs with *p* < 0.05 and the absolute value of the correlation coefficient >0.4 were screened out.

### Statistical Analysis

Univariate and multivariate Cox regression models were used to evaluate the predictive value of the ICI cohort’s potential biomarkers. Fisher’s exact test was used to evaluate the differences in the proportion of patients achieving complete response (CR)/partial response (PR) and progressive disease (PD)/stable disease (SD) between groups. The differences in gene mutation rates between groups were also evaluated by Fisher’s exact test. Wilcoxon’s test was employed to evaluate the differences in glycogen metabolism scores, immune cell proportions, TME-related signature scores, drug sensitivity between groups. Normally distributed data were tested using the unpaired *t*-test. Correlation analysis was used to evaluate the degree of correlation between the glycogen metabolism score and cell proliferation, wound healing, TGF-β response, TMB, TNB, and the proportion of immune cells. GSEA analysis was used to calculate the enrichment score and *p*-value of each pathway (Subramanian et al., 2005). The above analysis used R4.0.2 software for the statistical analyses and for compiling the figures.

## Results

### Glycogen Metabolism Scores Can be Used as an Independent Predictor of the Immunotherapy Efficacy in Urothelial Cancer patients.

As shown in Figure 1, our study contains two clinical cohorts: the ICI cohort published by Mariathasan et al., and the other is the TCGA cohort. To quantify the level of glycogen metabolism of UC patients, the ssGSEA algorithm was utilized to construct a glycogen metabolism score (GMS) based on the transcriptome data of the two cohorts. The ICI cohort data was used to explore the correlation between glycogen metabolism level and the ICI efficacy, while the data of both cohorts were prepared for subsequent exploration of the underlying mechanism.

**FIGURE 2:**
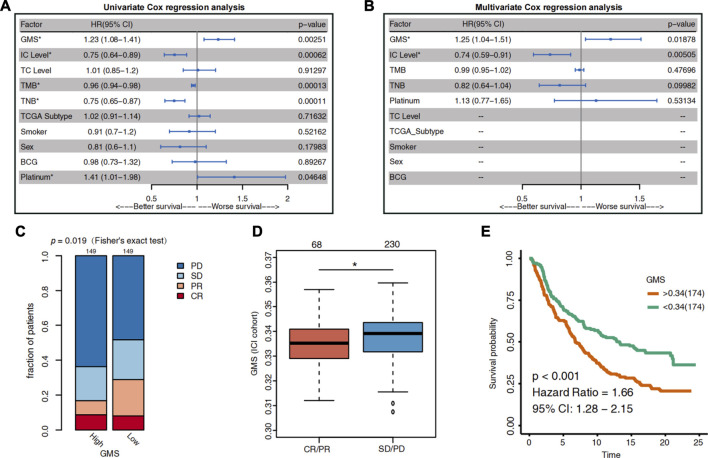
The predictive value of glycogen metabolism for the efficacy of ICI in UC patients. **(A)** The Forest plot shows the result of univariate Cox regression analysis. The indicators with *p* < 0.05 were GMS, IC level, TMB, TNB and platinum treatment. **(B)** The Forest plot visualizes the results of multivariate Cox regression analysis. Results showed that GMS and IC level were independent predictors of ICI in UC patients. The main portion of the plot depicts the hazard ratio (HR) and 95% confidence interval (95% CI). The statistically significant indicators (*p* < 0.05) are marked with asterisks after their name, and HR indicates whether the indicator portends a favorable prognosis (HR < 1) or a poor prognosis (HR > 1). **(C)** Differences in the proportion of UC patients with different response to ICI between high-GMS and low-GMS patients in the ICI cohort. CR: complete response; PR: partial response; PD: progressive disease; SD: stable disease. **(D)** Differences in GMS between CR/PR and SD/PD patients. The asterisk above the box plot indicates the range of *p* values. “.”: *p* < 0.1; “*”: *p* < 0.05; “**”: *p* < 0.01; “***”: *p* < 0.001. **(E)** Kaplan-Meier survival curves for OS in high-GMS (*n* = 174) and low-GMS (*n* = 174) patients of ICI cohort.

In the previous literature search, we found that the PD-L1 expression level (Rosenberg et al., 2016; Balar et al., 2017; Powles et al., 2017; Sharma et al., 2017), TMB (Rosenberg et al., 2016; Powles et al., 2018), TNB (Huang et al., 2018a; Chen et al., 2019; Raimondi et al., 2020), and TCGA subtypes (Rosenberg et al., 2016; Sharma et al., 2017) were potential biomarkers for predicting the ICI efficacy in UC patients. Considering the impact of the patients’ other treatments on the ICI efficacy, we integrated several factors into the analysis using the univariate COX regression model. These included the potential prognostic indicators, the other treatments patients received, and the clinical information of the ICI cohort (Figure 2A). Simultaneously, the statistically significant biomarkers (*p* < 0.05) were incorporated into the multivariate Cox regression analysis (Figure 2B). Two metrics quantify the expression level of PD-L1. One is the expression level of PD-L1 on tumor cells (TC), and the other is the expression level of PD-L1 on immune cells (IC).

The results of the multivariate Cox regression analysis showed that GMS [HR = 1.26 (95% CI 1.04–1.52), *p* = 0.017] and IC level [HR = 0.74 (95% CI 0.6–0.92), *p* = 0.006] were the independent predictors of prognosis for UC patients receiving ICI treatment (Figure 1B). The higher the GMS score, the worse the prognosis of immunotherapy for UC patients. Consistent with the results of the IMvigor210 trial, the IC level was a positive predictor of ICI efficacy (HR <1); i.e., the higher the IC level, the better the survival of UC patients after receiving immunotherapy.

To clarify the relationship between GMS and the efficacy of immunotherapy in UC patients, we divided the patients into high-GMS and low-GMS groups based on their median GMS. Then we compared the proportion of patients with different ICI responses between the two groups. In the ICI cohort, we found that, compared to low-GMS patients, the high-GMS patient group had a lower proportion of patients with CR and PR and a higher proportion of patients with PD and SD to ICI (two-tailed Fisher’s exact test, *p* = 0.019, Figure 2C). The GMS of the CR/PR cluster was also lower than that of the SD/PD cluster (Wilcoxon test, *p* = 0.0196, Figure 2D). Moreover, the results of the KM survival analysis of the ICI cohort showed that high-GMS patients exhibited a shorter OS compared to low-GMS patients (log-rank test, HR = 1.66 [95% CI:1.28–2.15], *p* < 0.001, Figure 2E). Although the result of KM survival analysis of the TCGA cohort had the same trend, the *p*-value was not significant [HR = 1.26 (95% CI: 0.94–1.69), *p* = 0.117, Supplementary Figure S1A], indicating that GMS may reflect the prognosis of patients treated with ICI. In addition, we found that GMS was positively correlated with proliferation (*p* = 0.019, *r* = 0.12, Supplementary Figure S1B) and wound healing (*p* < 0.001, r = 0.21, Supplementary Figure S1C) in the TCGA cohort, suggesting tumor malignancy and the possibility of a worse prognosis.

### Analysis of Genomics

**FIGURE 3:**
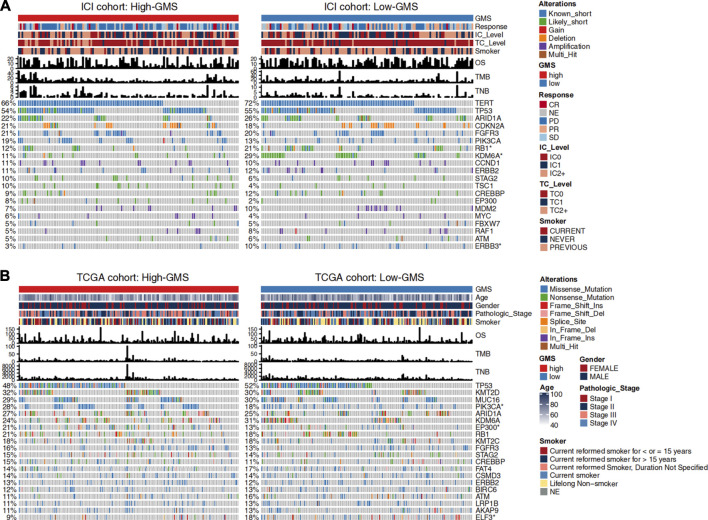
Clinical information and gene mutation landscape of UC patients in the ICI cohort **(A)** and TCGA cohort **(B)**. The main portion of the figure shows the top 20 driver genes’ alteration types with the highest mutation rates, and the left bar plot indicates the mutation rate of each driver gene. Genes mutated significantly between high-GMS and low-GMS patients are marked with asterisks after their name. The upper bar plot shows GMS subgroups, OS, TMB, TNB, and other clinical information of UC patients, and the color codes and annotations are shown in the legend.

Changes in disease phenotypes are attributed to changes in genomics. In order to explore the related underlying mechanisms of glycogen metabolism and ICI efficacy, we compared the gene mutation rates between high-GMS and low-GMS patients (Figure 3). The gene mutation landscape showed the alteration types and frequencies of the top 20 driver genes in ICI and TCGA cohorts. We obtained a total of six genes with significant differences in mutation frequencies among high-GMS and low-GMS patients (Figure 3), which were *RB1* (12 vs 21%), *KDM6A* (11 vs 29%), *ERBB3* (3 vs 10%) in the ICI cohort; and *PIK3CA* (28 vs 18%), *EP300* (21 vs 13%), *ELF3* (9 vs 18%) in the TCGA cohort. The complete results of comparisons of the gene mutation frequencies between high-GMS and low-GMS patients are provided in Supplementary Table S1.

To further explore the role of gene mutations in the effect of glycogen metabolism on ICI efficacy, we divided UC patients into mutant and wild-type groups based on the mutation status of the six genes screened and compared the differences of GMS and ICI efficacy between groups. In the ICI cohort, *KDM6A*-mutant (Wilcoxon test, *p* = 0.00057, Supplementary Figure S2B) and *ERBB3*-mutant (Wilcoxon test, *p* = 0.0085, Supplementary Figure S2C) patients had lower GMS than wild-type patients, while *PIK3CA*-mutant (Wilcoxon test, *p* = 0.04, Supplementary Figure S2D) and *EP300*-mutant patients (Wilcoxon test, *p* = 0.018, Supplementary Figure S2E) had opposite results (results for the *ELF3* gene were not shown because *ELF3* was absent in the sequencing data of the ICI cohort). The result for *RB1* gene was not significant (Wilcoxon test, *p* = 0.12, Supplementary Figure S2A) in the ICI cohort. Subsequently, we compared the differences in ICI efficacy between patients with mutant and wild-type genes. The results for *RB1*, *ERBB3* and *PIK3CA* genes were non-significant (two-tailed Fisher’s exact test, *p* > 0.05, Supplementary Figure S2F), while the proportion of patients achieving CR/PR was higher in *KDM6A*-mutant (two-tailed Fisher’s exact test, *p* = 0.028, Supplementary Figure S2F) and *EP300*-mutant (two-tailed Fisher’s exact test, *p* = 0.046, Supplementary Figure S2F) patients when compared to wild-type patients. Similarly, in the TCGA cohort, we also explored the differences in GMS, with results suggesting non-significant differences for *RB1*, *ERBB3* and *EP300* (Wilcoxon test, *p* > 0.05, Supplementary Figures S2G,I,K), lower GMS in patients with *KDM6A* (Wilcoxon test, *p* = 0.008, Supplemntary Figure S2H) and *ELF3* (Wilcoxon test, *p* = 0.016, Supplementary Figure S2L) mutations than in wild-type patients, and opposite results for *PIK3CA* (Wilcoxon test, *p* = 0.0089, Supplementary Figure S2J) genes.

In addition to gene mutation frequency, the distribution of various UC patients’ clinical information between high-GMS and low-GMS groups can also be observed. Results suggested no significant correlation between GMS and TMB or TNB in both cohorts (Figure 3, Supplementary Figures S1D–G). Although smoking is a risk factor for bladder cancer (Saginala et al., 2020), smoking or not smoking had no prognostic value in the univariate Cox regression analysis [HR = 0.91 (95% CI 0.7–1.2), *p* = 0.522, Figure 2A]. GMS distribution was also not statistically significant between smokers and non-smokers (Wilcoxon test, *p* = 0.39, Supplementary Figure S1H). Among the four molecular TCGA subtypes, TCGA subtype III had the highest GMS, while TCGA subtype II had the lowest GMS (Supplementary Figure S1I).

### Immune Infiltration Landscape

**FIGURE 4:**
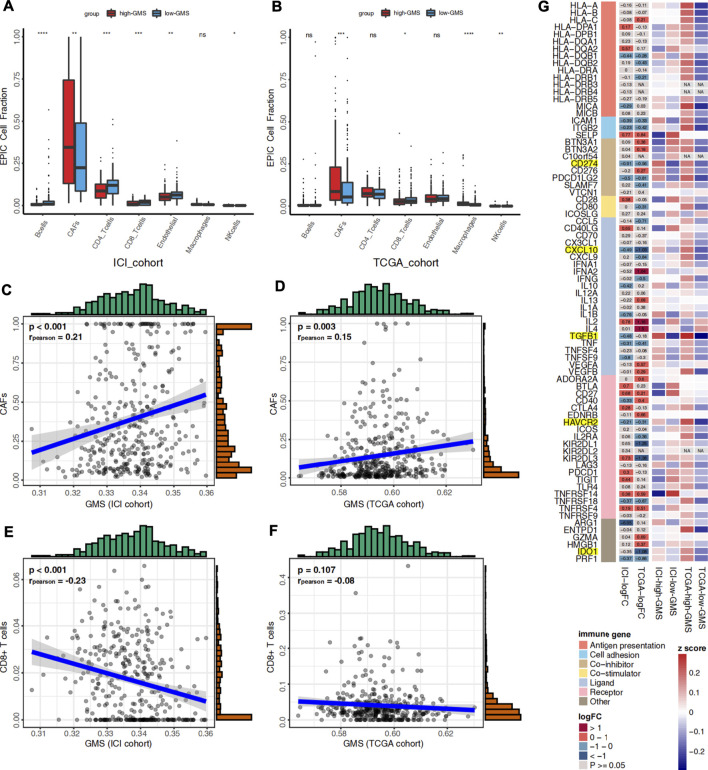
**(A)** Box plot visualizes the proportion of 7 cell types in the tumor microenvironment between high-GMS and low-GMS patients in the ICI cohort. **(B)** Box plot visualizes the proportion of 7 cell types in the tumor microenvironment between high-GMS and low-GMS patients in the TCGA cohort. **(C)** The correlation between GMS and the proportion of CAFs in the ICI cohort. **(D)** The correlation between GMS and CAFs proportion in the TCGA cohort. **(E)** The correlation between GMS and the proportion of CD8^+^ T cells in the ICI cohort. **(F)** The correlation between GMS and the proportion of CD8^+^ T cells in the TCGA cohort. **(G)** The Heatmap shows the expression levels of immune-related genes and immune exhaustion biomarkers between patients with high-GMS and low-GMS. The color in the first column of the heatmap represents the classification of the molecules according to their immune functions, and the meaning of the colors is shown in the legend. The second and third columns exhibit the logFC and *p*-value of the analysis of the gene differential expression analysis. The color represents the size of logFC shown in the middle of the rectangles, while gray represents non-significant *p*-values. In the calculation of logFC, high-GMS patients were used as the control group. LogFC >0 means that the genes were highly expressed in low-GMS patients, while logFC <0 is the opposite. The last four columns of the heatmap show the average gene expression levels, which were standardized by z-score.

Like tumor cells, immune cells and stromal cells also play an important role in the tumor immune microenvironment. Through its association with energy metabolism, glycogen metabolism is important for tumor cells and has an essential impact on immune cells. Using the EPIC algorithm, we analyzed the differences in the proportions of immune and stromal cells between high-GMS and low-GMS patients. An elevated proportion of tumor-associated fibroblasts (CAFs) and a reduced proportion of CD8^+^ T cells can be observed in high-GMS patients (Figures 4A,B). Consistent with the results above, correlation analysis also revealed a positive correlation between the proportion of CAFs and GMS (ICI cohort: *p* < 0.001, *r* = 0.21; TCGA cohort: *p* = 0.003, *r* = 0.15, Figures 4C,D). In contrast, the proportion of CD8^+^ T cells was negatively correlated with GMS (ICI cohort: *p* < 0.001, *r* = −0.23; TCGA cohort: *p* = 0.107, *r* = −0.08, Figures 4E,F).

To further reveal the relationship between glycogen metabolism and the immune microenvironment at the genomic level, we analyzed the differences in the expression of immune-related genes and immune exhaustion biomarkers between high-GMS and low-GMS patients. In both cohorts, we found that TGFβ, CXCL10, PD-L1 (CD274), and other immunosuppressive molecules, including HAVCR2 and IDO1, were elevated in high-GMS patients (Figure 3G). In addition, we explored TGF-β′s effect on the ICI efficacy in our TCGA cohort; we also found that TGF-β was positively correlated with GMS (TCGA cohort: *p* < 0.001, *r* = 0.38, Supplementary Figure S1J).

To further clarify the effect of glycogen metabolism on the immune microenvironment, we compared the changes in the TME-related signatures between GEO-control and GEO-KO samples. As a resident enzyme of the endoplasmic reticulum, glucosidase II plays an important role in glucose metabolism and glycoprotein quality control (Kuribara et al., 2020). As shown in Supplementary Figure S3A,B, the GMS of the GEO-KO samples were significantly lower than those of GEO-control samples (*t*-test, *p* = 0.041, Supplementary Figure S3A,B). Based on the median GMS of the six samples, we classified three samples from the control group into the high-GMS group and three samples from the GEO-KO group into the low-GMS group. Among the 119 TME-related signatures, we found that signatures related to myeloid-derived suppressor cell (MDSC) and neutrophils demonstrated a consistent trend across three datasets. High-GMS patients and GEO-control samples had higher MDSC (ICI cohort: Wilcoxon test, *p* < 0.01; TCGA cohort: Wilcoxon test, *p* < 0.01; GSE164042: Wilcoxon test, *p* = 0.018, Supplementary Figure S3C–E) and neutrophil (ICI cohort: Wilcoxon test, *p* < 0.01; TCGA cohort: Wilcoxon test, *p* < 0.01; GSE164042: Wilcoxon test, *p* = 0.012, Supplementary Figure S3F–H) signature scores compared to low-GMS patients and GEO-KO samples.

### Gene Set Enrichment Analysis

**FIGURE 5:**
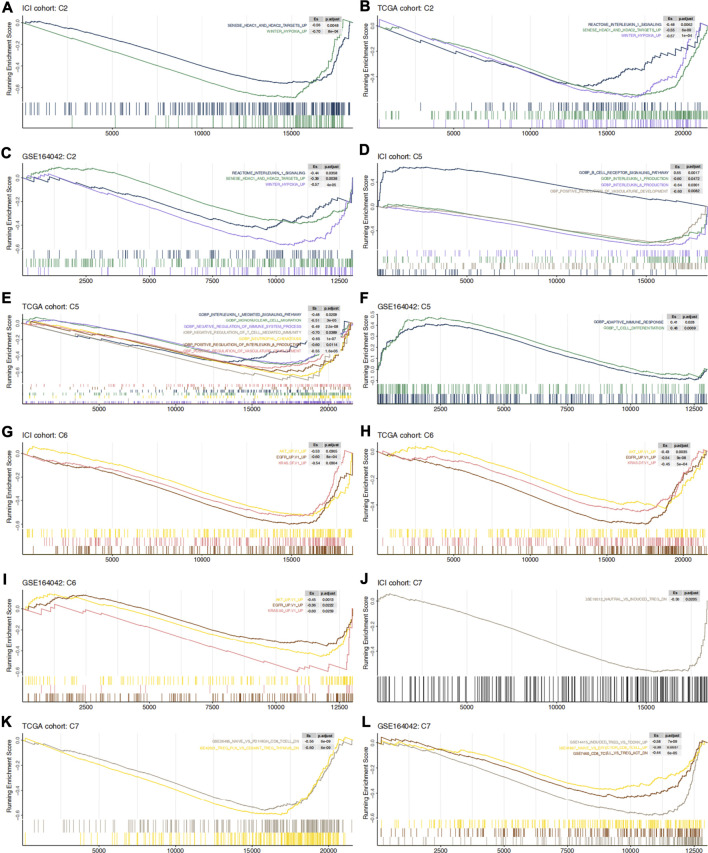
C2 **(A–C)**, C5 **(D–F)**, C6 **(G–I)** and C7 **(J–L)** gene set enrichment analysis in ICI cohort, TCGA cohort and GSE164042 samples. High-GMS patients and GEO-control group served as the control group. ES >0 means that the corresponding pathway is significantly enriched in low-GMS patients or GEO-KO samples, while ES <0 is the opposite.

The GSEA algorithm can help determine whether glycogen metabolism crosstalks with the immune microenvironment through some abnormally activated signaling pathways. Hypoxia as well as histone deacetylase (HDAC) related pathways were mainly enriched in high-GMS patients and GEO-control samples (Figures 5A–C). Consistent with the results above, monocyte and neutrophil recruitment, Treg-related pathways, immune-depleted CD8^+^ T cells, and immunosuppressive cytokines (such as IL-1 and IL-8) were predominantly enriched in high-GMS patients and GEO-control samples (Figures 5C–E,J–L). In contrast, T cells and B cells signaling pathways associated with immune activation were mainly enriched in low-GMS patients and GEO-KO samples (Figures 5D–F,K,L). In addition, angiogenesis and classical carcinogenic pathways (such as AKT, EGFR, KRAS signaling pathways) were also enriched in high-GMS patients and GEO-control samples (Figures 5E,G–I). The statistically significant results of GSEA analysis are available in the Supplementary Table S2.

### Prediction of Drug Sensitivity

Predicting drug sensitivity helps us promote the translation from research discovery to clinical application and provides a new perspective for potential treatment strategies. We predicted the sensitivity of UC patients to various drugs based on the public data of CLUE, CTRP, CCLE databases, and the RNA expression profiles of our clinical cohorts. Mode-of-action (MoA) analysis helps us to summarize the mechanism of action of the screened drugs.

**FIGURE 6:**
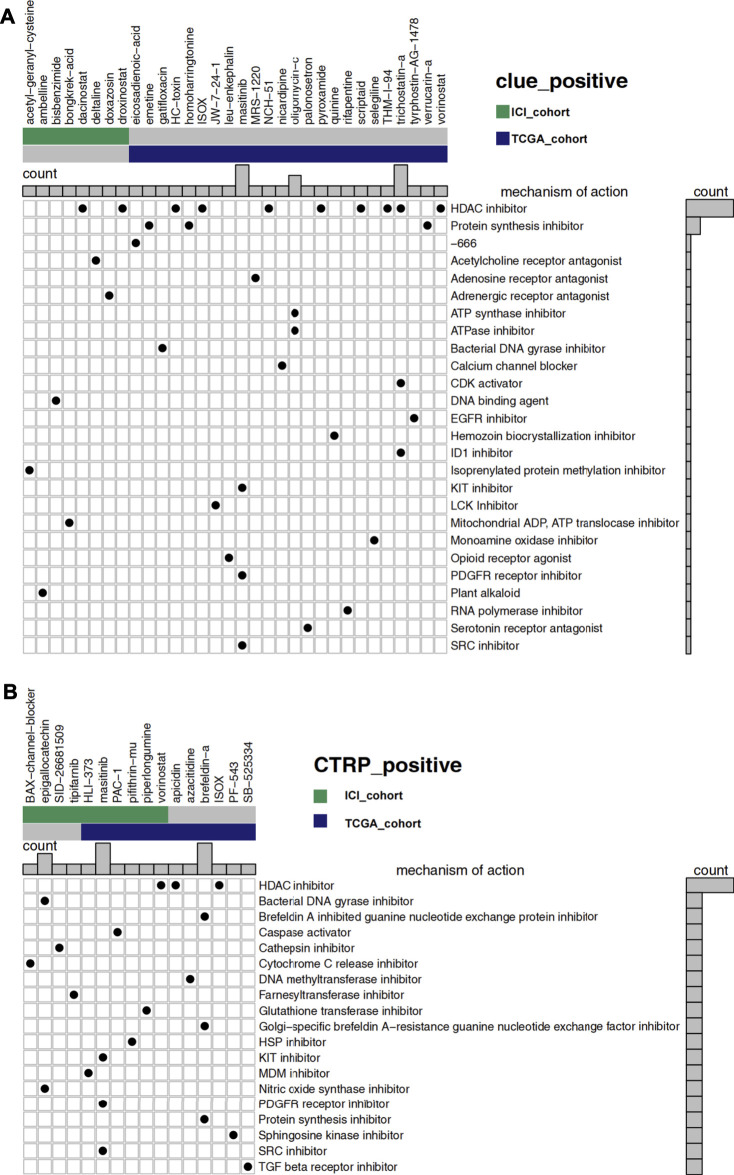
**(A)** The degree of similarity between the expression profiles of UC patients and that of cell lines treated with different drugs. In the analysis of the CLUE database, high-GMS patients were used as the control group, and a positive value represents a similar trend while a negative value represents an opposite trend. The similarity is quantified with a score of −100 to 100. The higher the absolute value of the score, the more obvious the trend. The figure shows drugs with scores above 60. **(B)** The predicted sensitivity of UC patients to various drugs using data from the CTRP and CCLE databases while the drug sensitivity is quantified with AUC. The lower the AUC value, the higher the sensitivity to the drug. The figure shows the drugs whose AUC values were significantly different between high-GMS and low-GMS patients (Wilcoxon’s test, *p* < 0.05). In addition, the correlation coefficients of the drugs above with GMS were greater than 0.4, which indicated that these drugs were more sensitive in patients with low-GMS. In the main portion of the figure, columns represent drugs, and rows represent the mechanisms of action. The black dots in the corresponding rectangles indicate that the drugs have the corresponding mechanisms of action. The bar plots on the top and right of the figure reflect the frequency of black dots of the corresponding columns/rows.

Figure 6A showed 32 drugs with a score greater than 60 in the result of the CLUE database analysis. After the treatment of these drugs, the RNA expression profiles of the cell lines were similar to that of low-GMS patients, implying that these drugs may reverse the low susceptibility of high-GMS patients to ICI. MoA analysis of the 32 compounds revealed 26 mechanisms of action. Figure 6B showed the analysis results of the CTRP database, and 16 drugs were screened out. The 16 drugs were statistically significant in the Wilcoxon test (*p* < 0.05), and their correlation coefficients with GMS were greater than 0.4, suggesting that low-GMS patients were more sensitive to these drugs. MoA analysis of the 16 drugs revealed their 19 mechanisms of action. Among the drugs mentioned above, we noticed that there were 11 HDAC inhibitors totally (dacinostat, droxinostat, HC-toxin, ISOX, NCH-51, pyroxamide, scriptaid, THM-I-94, trichostatin-a, vorinostat, and apicidin, respectively, Figures 6A,B). Researchers have revealed the potential anti-tumor activity of dacinostat (Ganai, 2015), droxinostat (Huang et al., 2018b), HC-toxin (Zhou et al., 2016), NCH-51 (Sanda et al., 2007), pyroxamide (Butler et al., 2001), scriptaid (Takai et al., 2006), trichostatin-a (Vigushin et al., 2001), vorinostat (Gray et al., 2019; Rodriguez et al., 2020) and apicidin (Ueda et al., 2007), indicating that the combination treatment of immunotherapy and HDAC inhibitors in UC patients may achieve better clinical outcomes.

Finally, we identified 32 drugs with a score less than -60 due to the CLUE database analysis (Supplementary Figure S2A). After the treatment with these drugs, the RNA expression profiles of the cell lines were similar to that of high-GMS patients. The four MoA mechanisms that contain the most screened drugs include the adrenergic receptor antagonist, glucocorticoid receptor antagonist, prostanoid receptor antagonist, and tubulin inhibitor, which may be detrimental to the response of UC patients to immunotherapy. Under the constraints of *p* < 0.05 (Wilcoxon test) and correlation coefficient < −0.4, we screened out five drugs in the analysis of the CTRP database (Supplementary Figure S2B). The results suggested that high-GMS patients were more sensitive to these drugs when compared to low-GMS patients. MoA analysis of the five drugs also revealed their 11 mechanisms of action. We observed that some of these five drugs targeted apoptosis, PI3K, and RAF signaling pathways, which provided novel insight into the treatment of high-GMS patients. In particular, betulinic-acid impacts multiple signaling pathways as a topoisomerase inhibitor, showing its prospect for further investigation.

## Discussion

Since immunotherapy resolved the treatment dilemma of urothelial cancer over the past 30 years, ICI has brought new sight into the treatment of UC patients. In order to promote the development of precision medicine, we need to screen for more potential biomarkers to identify ICI-sensitive UC patients. Our results showed that glycogen metabolism could be used as an independent predictor of UC patients’ prognosis. Low-GMS patients responded better to ICI treatment, with a lower proportion of CAFs cells and a higher proportion of CD8^+^ T cells. The GSEA analysis result suggested that immunosuppressive cells related pathways, along with the secretion of immunosuppressive cytokines, were mainly enriched in high-GMS patients. Based on analysis of the drug sensitivity data, we speculated that the combination of HDAC inhibitor and ICI might improve the efficacy of immunotherapy in UC patients. High-GMS patients may choose drugs targeting apoptosis, such as topoisomerase inhibitors, as potential therapeutic approaches.

Among the top 20 driver genes exhibiting the most frequent mutation rates in our clinical cohorts, we found six genes with significant differences between high and low GMS patients (Figure 3). Compared to high-GMS patients, *RB1*, *KDM6A*, *ERBB3*, and *ELF3* had higher mutation rates in low-GMS patients, while *PIK3CA* and *EP300* had lower rates. *EP300* was identified as a co-activator of hypoxia-inducible factor 1α (HIF1A), which plays a vital role in activating hypoxic responses (Wei et al., 2018). Due to the use of anti-angiogenic drugs or the fast tumor growth rate, hypoxia is an important feature of the tumor microenvironment (Samanta and Semenza, 2018); it contributes to the increase of synthesis of hypoxia-inducible factor (HIF). Under the induction of HIF, glycogen synthesis and catabolism enzymes are elevated, contributing to the survival and metastasis of tumor cells under nutrient deprivation (Samanta and Semenza, 2018). Consistent with these investigations, our results showed that *EP300* mutated most frequently in high-GMS patients. This trend suggests that the hypoxic microenvironment may affect the level of tumor cell glycogen metabolism through *EP300* mutation in UC patients. Our GSEA results also showed that hypoxia-related pathways mainly enriched in high-GMS patients and GEO-control samples (Figures 5A–C). Reports indicate that the oncogenes, *ERBB3* (Sithanandam et al., 2003; Sithanandam et al., 2005), *ELF3* (Wang et al., 2018; Zhao et al., 2018; Zhang et al., 2020), and *PIK3CA* (German et al., 2013), activate the PI3K-AKT pathway either directly or indirectly. Also, the activation of the PI3K-AKT pathway can negatively regulate GSK3β (Sithanandam et al., 2003); thus, its activation inhibits the activity of glycogen synthase and reduces glycogen synthesis (Khan et al., 2020). However, the distributions of the mutation status of *ERBB3*, *ELF3*, and *PIK3CA* between high-GMS and low-GMS patients were not consistent, indicating that multiple biological processes regulated the level of glycogen metabolism, and the underlying mechanisms need further exploration at a genetic level.

The immune microenvironment, consisting of tumor cells, immune cells, stromal cells, vasculature, cytokines, and chemokines, has always focused on immunotherapy research. During immunotherapy, the competition between tumor cells and other cells determines the treatment’s efficacy in UC patients. We found that levels of CAFs were higher in high-GMS patients through the EPIC algorithm. Studies have shown that TGF-β released by tumor cells activated the p38-MAPK pathway in CAFs, increasing the levels of chemokines such as CXCL10, IL-6, and IL-8, thereby inducing glycogen catabolism in tumor cells (Coller, 2019). Notably, this trend is consistent with the expression of immune-related genes and the result of GSEA analysis in our study. IL-1 related pathways, which were enriched in high-GMS patients in the GSEA analysis, help to maintain the phenotype of CAFs and enhance their function (Chen and Song, 2019). IL-6 and IL-8 secreted by CAFs can promote the differentiation of myeloid cells into MDSCs or M2 macrophages to assist tumor immune escape (Kim et al., 2012). CAFs also express the FAS ligand (FASL), which induces apoptosis in FAS-expressing CD8^+^ T cells (Lakins et al., 2018). Our research also suggested that the proportion of CD8^+^ T cells in high-GMS patients was significantly lower than that of low-GMS patients (Figures 4A,B). In addition, CAFs are also the major source of proangiogenic factors, supporting angiogenesis in the tumor microenvironment (Unterleuthner et al., 2020; Du et al., 2017). In addition to CAFs, GSEA analysis also revealed that the recruitment of monocytes and neutrophils and Treg related pathways were also enriched in high-GMS patients (Figures 5E,J–L). Numerous studies have shown that monocytes may undergo differentiation and become M2 macrophages (Cho et al., 2018), and neutrophils could differentiate into MDSCs in the tumor microenvironment (Giese et al., 2019). M2 macrophages (Wen et al., 2018; Zhu et al., 2020) and neutrophils (Zhao et al., 2020) play an immunosuppressive role in the elimination of tumor cells. After comparing the differences in TME-related signature scores, we also found that MDSC and neutrophil signature scores of GEO-KO samples were lower than those of GEO-control samples (Supplementary Figures S3C–H).

The application of ICI has profoundly changed the strategies of treatment for urothelial carcinoma. However, only 20–30% of UC patients respond to immunotherapy, and some still have the problem of primary or secondary resistance. In addition, to identify patients sensitive to immunotherapy, the development of novel drug combinations may help us enhance the efficacy of immunotherapy. By analyzing the drug sensitivity data, we speculated that HDAC inhibitors might enhance ICI efficacy and even reverse the poor response of low-GMS patients to ICI treatment, which was consistent with our GSEA results (Figures 5A–C). Some studies have reported HDAC inhibitors’ potential in enhancing the efficacy of immunotherapy and even reverse the insensitivity to ICI (Ugurel et al., 2019) by enhancing the anti-tumor activity of CD8^+^ T cells (Que et al., 2021) and NK cells (Hicks et al., 2018; Kim et al., 2020), reducing the number of M2 macrophages (Knox et al., 2019; Kim et al., 2020), impairing the immunosuppressive function of MDSCs (Kim et al., 2020; Que et al., 2021) and increasing the expression of PD-L1 on tumor cells (Hicks et al., 2018; Que et al., 2021). Two clinical studies investigated the efficacy of HDAC inhibitor vorinostat, combined with immunotherapy, demonstrating preliminary antitumor activity (Gray et al., 2019; Rodriguez et al., 2020). In addition, we also screened out five drugs with high sensitivity in high-GMS patients, which target apoptosis, PI3K, and RAF signaling pathways. The results obtained from the genomic analysis indicated that multiple significantly mutated genes have regulatory effects on the PI3K signaling pathway; consequently, the exploration of drugs targeting the PI3K signaling pathway may be a new direction for the combined treatment of UC patients. Since there are relatively few clinical studies on the combination of ICI and other drugs in UC patients, we need more prospective studies to provide strong evidence for novel drug combinations.

In this study, the bioinformatic analysis of two clinical cohorts helped us reveal the prognostic value of glycogen metabolism on ICI efficacy in UC patients as well as its possible underlying mechanism. However, there are still several limitations. First, the results of our study are based on the investigation of only one ICI cohort and TCGA cohort; more data from other ICI cohorts would produce more convincing, robust results. Secondly, we utilized the RNA-seq data to construct the GMS of UC patients. The RNA-seq data represents RNA levels of the mixtures of various cell types in the tumor microenvironment. Although GMS mainly represented glycogen metabolism levels of tumor cells, this data is also combined with the metabolic levels of immune cells and stromal cells. In addition, the specific mechanism of glycogen metabolism’s effect on the tumor microenvironment requires more research and experimental evidence.

## Data Availability

The datasets presented in this study can be found in online repositories. The names of the repository/repositories and accession number(s) can be found in the article/Supplementary Material.
