# Supplementary material for: Glycogen Metabolism Predicts the Efficacy of Immunotherapy for Urothelial Carcinoma
Source: Front Pharmacol. 2021 Aug 25;12:723066. doi: 10.3389/fphar.2021.723066 (PMC8424112; doi:10.3389/fphar.2021.723066)
Supplement: Supplementary file 2 [file DataSheet1.pdf]

Supplementary Material

Supplementary Figures

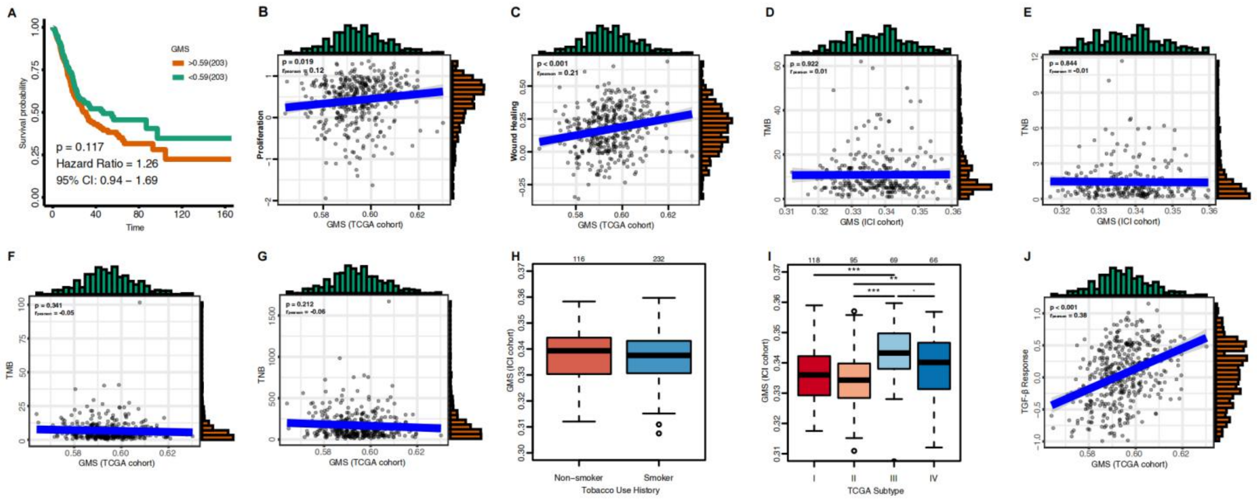

Supplementary Figure 1. (A) Kaplan-Meier survival curves for OS in high-GMS (n = 203) and low-GMS (n = 203) patients of the TCGA cohort. (B) The correlation between GMS and proliferation score in the TCGA cohort. (C) The correlation between GMS and wound healing score in the TCGA cohort. (D) The correlation between GMS and TMB in the ICI cohort. (E) The correlation between GMS and TNB in the ICI cohort. (F) The correlation between GMS and TMB in the TCGA cohort. (G) The correlation between GMS and TNB in the TCGA cohort. (H) The distribution of GMS between smokers and non-smokers. (I) The distribution of GMS in different TCGA molecular subtypes. Asterisks above the box plot indicate the range of p values. ".": P < 0.1; "*": p < 0.05; "**": p < 0.01 ;"***": p < 0.001. (J) The correlation between GMS and TGF-β response in the TCGA cohort.

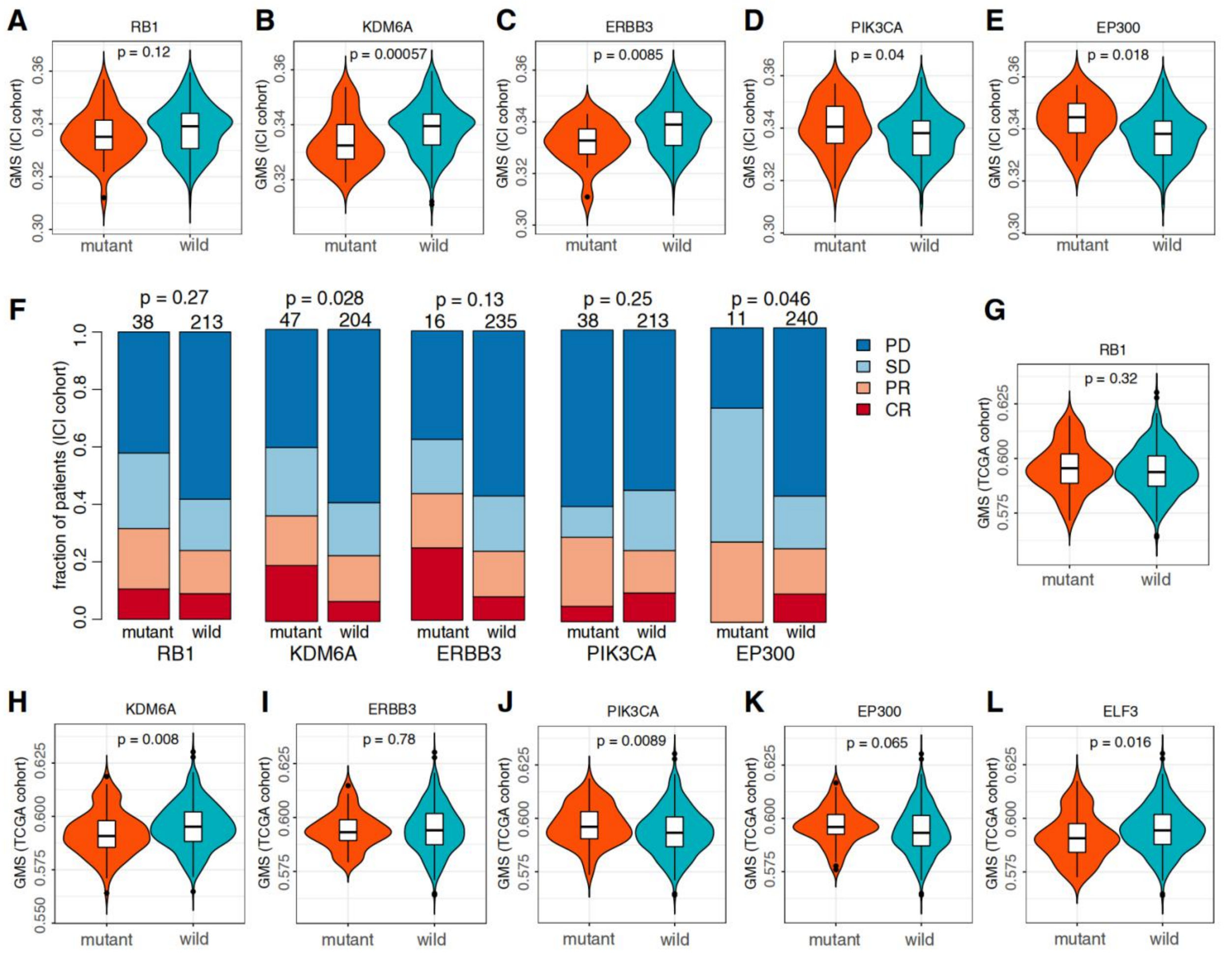

Supplementary Figure 2. (A) Differences in GMS between RB1-mutant and RB1-wild patients in the ICI cohort. (B) Differences in GMS between KDM6A-mutant and KDM6A-wild patients in the ICI cohort. (C) Differences in GMS between ERBB3-mutant and ERBB3-wild patients in the ICI cohort. (D) Differences in GMS between PIK3CA-mutant and PIK3CA-wild patients in the ICI cohort. (E) Differences in GMS between EP300-mutant and EP300-wild patients in the ICI cohort. (F) Differences in the proportion of UC patients with different responses to ICI between gene-mutant and gene-wild patients in the ICI cohort. CR: complete response; PR: partial response; PD: progressive disease; SD: stable disease. (G) Differences in GMS between RB1-mutant and RB1-wild patients in the TCGA cohort. (H) Differences in GMS between KDM6A-mutant and KDM6A-wild patients in the TCGA cohort. (I) Differences in GMS between ERBB3-mutant and ERBB3-wild patients in the TCGA cohort. (J) Differences in GMS between PIK3CA-mutant and PIK3CA-wild patients in the TCGA cohort. (K) Differences in GMS between EP300-mutant and EP300-wild patients in the TCGA cohort. (L) Differences in GMS between ELF3-mutant and ELF3-wild patients in the TCGA cohort.

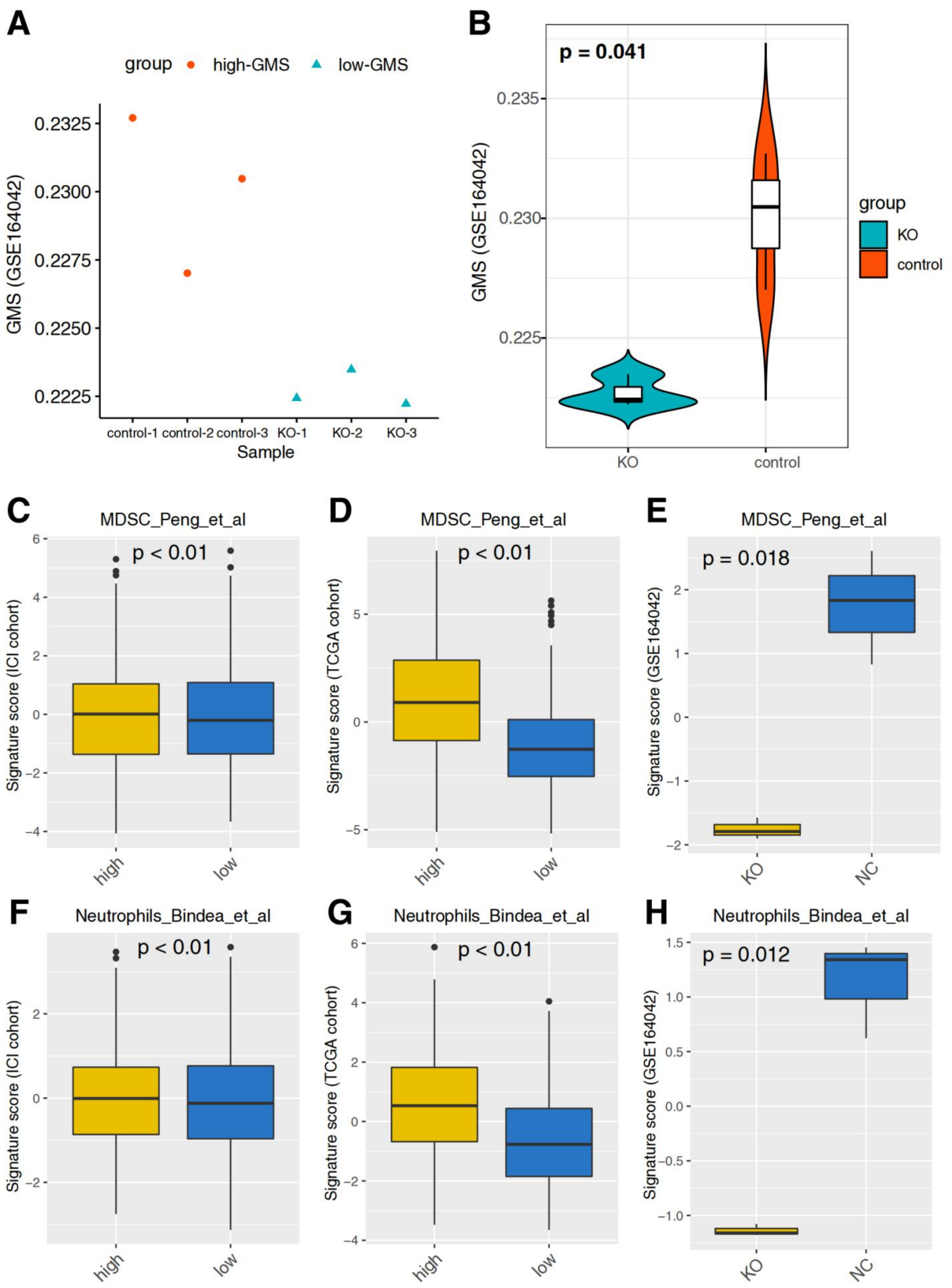

Supplementary Figure 3. (A) Scatter plot showing the GMS of six samples in the GSE164042 dataset. The six samples were divided into two groups, high-GMS and low-GMS, according to their median GMS. (B) Box plot showing the differences in GMS between GEO-control and

GEO-KO samples. (C) Box plot showing the differences in the MDSC-related signature scores between high-GMS and low-GMS patients in the ICI cohort. (D) Box plot showing the differences in the MDSC-related signature scores between high-GMS and low-GMS patients in the TCGA cohort. (E) Box plot showing the differences in the MDSC-related signature scores between GEO-control and GEO-KO samples. (F) Box plot showing the differences in the neutrophil-related signature scores between high-GMS and low-GMS patients in the ICI cohort. (G) Box plot showing the differences in the neutrophil-related signature scores between high-GMS and low-GMS patients in the TCGA cohort. (H) Box plot showing the differences in the neutrophil-related signature scores between GEO-control and GEO-KO samples.

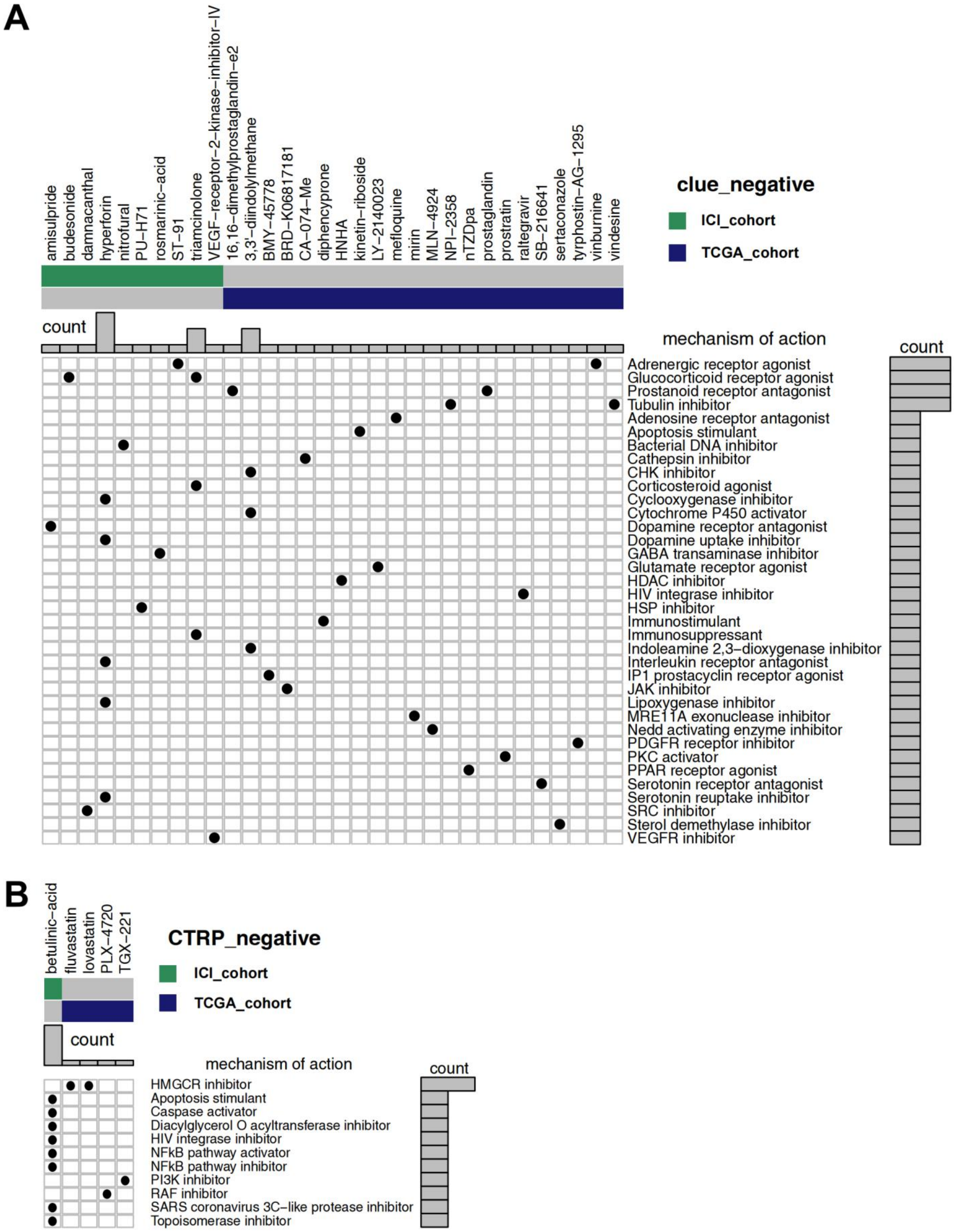

Supplementary Figure 4. (A) The degree of similarity between the expression profiles of UC patients and that of cell lines treated with different drugs. In the analysis of the CLUE database, high-GMS patients were used as the control group, and a positive value represents a similar trend while a negative value represents an opposite trend. The similarity is quantified with a score of -100 to 100. The higher the absolute value of the score, the more obvious the trend. The figure shows drugs with scores below -60. (B) The predicted sensitivity of UC patients to various drugs

using data from the CTRP and CCLE database and the drug sensitivity is quantified with the AUC. The lower the AUC value, the higher the sensitivity to the drug. The figure shows the drugs whose AUC values were significantly different between high-GMS and low-GMS patients (Wilcoxon's test, $p < 0.05$). In addition, the correlation coefficients of the drugs above with GMS were lower than -0.4, which indicated a higher sensitivity to these drugs in high-GMS patients. In the main portion of the figure, columns represent drugs, and rows represent the mechanisms of action; the black dots in the corresponding rectangles indicate that the drugs have the corresponding mechanisms of action. The bar plots on the top and right of the figure reflect the frequency of black dots of the corresponding columns/rows.
